# Accelerometer‐measured sleep behaviour and parent–child sleep guideline adherence and sleep quality in Czech families with children aged 3–8 years: the FAMIly Physical Activity, Sedentary behaviour and Sleep (FAMIPASS) study

**DOI:** 10.1111/jsr.14242

**Published:** 2024-05-16

**Authors:** Jaroslava Voráčová, Erik Sigmund, Michal Vorlíček, Jan Dygrýn, Dagmar Sigmundová

**Affiliations:** ^1^ Faculty of Physical Culture, Department of Social Sciences in Kinanthropology Palacký University Olomouc Olomouc Czech Republic; ^2^ Faculty of Physical Culture, Institute of Active Lifestyle, Faculty of Physical Culture Palacký University Olomouc Olomouc Czech Republic

**Keywords:** actigraphy, children, family, parents, preschooler, sleep

## Abstract

Assessing parent–child relationship in sleep behaviours is important for facilitating changes in the sleep guideline compliance in preschool age children. The aim of this study was to examine accelerometer‐measured sleep quantity and quality in families with children aged 3–8 years and investigate the parents’ influence on the child's sleep. The data were obtained from the Czech cross‐sectional FAMIly Physical Activity, Sedentary behaviour and Sleep (FAMIPASS) study, with a final sample of 374 families. Families were recruited through the enrolment of their children in kindergartens/primary schools between March 2022 and May 2023. The sleep time window and total sleep time were assessed using a wrist‐worn ActiGraph accelerometer. Participants wore this device continuously for 24 h/day over a period of 7 consecutive days. Demographic data and potential correlates were obtained via questionnaires completed by parents. Statistical analyses were completed using logistic regression and independent‐samples Mann–Whitney *U* test. In all, 65.5% of children (60% boys, 70.9% girls) and 58.3% of parents (52.4% fathers, 64.3% mothers) achieved the recommended sleep duration. Greater sleep quantity and duration in good‐quality sleep were significantly higher in girls/mothers, compared to boys/fathers. Preschoolers were more likely to comply with sleep guidelines if their mother (but not father) met the sleep recommendation and their mothers did not have a higher education level. Adhering to sleep guidelines in children was also associated with children's female gender, absence of screen device in the bedroom, and being more active. Given the high concurrence in mother–child sleep quantity, it is important to promote healthy sleep behaviours in the whole family.

## 1 INTRODUCTION

Adopting healthy behaviours, including sufficient sleep duration and quality, during childhood are essential for broad development and growth (El‐Sheikh & Sadeh, 2015) and may impact health and well‐being at a later age (Corepal et al., 2018; Quist et al., 2016). On the other hand, inadequate sleep in young children is linked with numerous health problems (e.g., overweight/obesity, emotional health problems, etc.) (Fairclough et al., 2023; Miller et al., 2020) and worse academic performance and decreased overall quality of life (Chaput et al., 2016). There is evidence that adherence to sleep guideline recommendations and good sleep hygiene in preschool and school‐age children are associated with numerous health benefits (Chaput et al., 2016; Quist et al., 2016) and play an important role in physical and mental health (Chaput et al., 2016). According to Canadian 24‐h movement guidelines children aged 3–4 years should sleep 10–13 h/24 h (including naps), and children aged 5–13 years 9–11 h/night (CSEP, n.d.), and these recommendations are similar to sleep guidelines by the American Academy of Sleep Medicine (AASM) and Sleep Research Society (Paruthi et al., 2016) or the World Health Organization (WHO, n.d.). Mixed findings have been observed in children's sleep duration (Decraene et al., 2021; Short et al., 2018). Results from a systematic review and meta‐analysis from 2016 including 23 studies on sleep duration and cognition showed that most of the children aged 5–13 years did not comply with AASM sleep guidelines (Short et al., 2018). In contrast, relatively high sleep recommendation adherence rates (69.2%) were observed in a recent study investigating sleep recommendation adherence, using the WHO 24‐h movement behaviour guidelines, related to adiposity via sleep questionnaires in preschoolers from six European countries (Belgium, Bulgaria, Germany, Greece, Poland and Spain; Decraene et al., 2021). Recent Czech data on sleep duration are available mostly for school‐aged children and adolescents (Gaba et al., 2020; Gariepy et al., 2020). A study published in 2020 showed that only 29% of Czech children aged 8–13 years slept for >9 h/night (Gaba et al., 2020).

Adequate sleep duration has been favourably associated with health in adults as well (Chaput et al., 2020). Canadian 24‐h movement guidelines recommend that adults should sleep 7–9 h/night (CSEP, n.d.). Only limited information is available about compliance with recommended sleep guidelines for adults and the data on sleep duration and its trends vary according to country of the research (Matricciani et al., 2017). United States data from the National Health Interview Survey reported that 59.8% of adults slept 7–8 h/night and 32.9% slept ≤6 h/night in 2017, and an increasing trend toward inadequate sleep duration was observed between 2013 and 2017 (Sheenan et al., 2019).

The family is an essential component in the child's development and plays a vital role in shaping behaviour and personality during childhood developmental stages (Rhodes et al., 2020; Tikotzky, 2017). Numerous studies revealed a positive relationship between parent–child interactions and health indicators in children, e.g., risk of childhood obesity (Niec et al., 2022); health literacy (Batool et al., 2024); and physical activity, sedentary, screen and sleep behaviours (Foo et al., 2022; Rhodes et al., 2020; Rollo et al., 2020). Based on evidence using mostly self‐reported measures and a few studies assessing sleep via accelerometer, significant associations between parents and their offspring were observed for sleep duration and quality/efficiency (Cimon‐Paquet et al., 2019; Kouros & El‐Sheikh, 2017; Varma et al., 2021; Varma et al., 2022). A recent (2022) systematic review reported seven studies that used actigraphy or electroencephalography to establish significant associations between parent–child sleep outcomes (Varma et al., 2021). These studies showed that sleep in parents/children is interrelated and that children's sleep duration and sleep efficiency are linked to their parents’ sleep, particularly mothers’ sleep (Kouros & El‐Sheikh, 2017; Varma et al., 2021). Parent–child sleep associations were also found in sleep disturbances and actigraphy data in a pilot study showed high concordance and night‐wakings between parents and children (Varma et al., 2022).

Positive parental support behaviour and bedtime routine are associated with improved compliance to sleep recommendations (McDowall et al., 2017; Philbrook et al., 2022). According to longitudinal design studies, multiple parental factors including parental bedtime habits and interactions, parental cognitions, maternal mood, stress, and co‐parenting were correlated with sleep of young children, which was in compliance with the transitional model of child sleep (Tikotzky, 2017). A study focusing on the relationship between parent–child sleep practices and problems in children aged 2–12 years reported that parents with higher education, higher annual income, and accurate estimation of their children's sleep needs were more likely to report that their children had earlier bedtimes and wake times, and more consistent sleep routines on weekdays and weekends (McDowall et al., 2017). Greater parent knowledge about child sleep (knowledge of healthy sleep guidelines and recommendations such as healthy sleep practices, bedtime routine, bedtime and wake times on week and weekend days, signs of sleep deprivation, and average sleep needs of children) obtained via questionnaire was also related with shorter sleep latencies in children (McDowall et al., 2017). In addition, greater parental involvement including higher parental sensitivity (parental acceptance, sensitivity, consideration, the quality of physical and vocal contact), quiet activities, and not using screen devices before bedtime were a predictor of longer child sleep duration and efficiency in children aged 3–6 years (Philbrook et al., 2022).

To date, no previous study has provided information on sleep behaviours in preschoolers from Central European countries (including the Czech Republic) using objective measures. Several studies have documented significant association between Czech parents and preschoolers for overweight/obesity, physical activity, and sedentary and screen behaviours (Sigmund et al., 2020; Sigmundova & Sigmund, 2021); however, very little is known about parent–child sleep duration and quality relationship. Therefore, the aims of this study were to examine the sleep behaviours (sleep duration, quality, and factors affecting sleep) of girls and boys from the Czech Republic aged 3–8 years and to investigate whether sleep guideline adherence in parents influence the odds of meeting sleep recommendations of their children.

## 2 METHODS

### 2.1 Study design and participants

**FIGURE 1:**
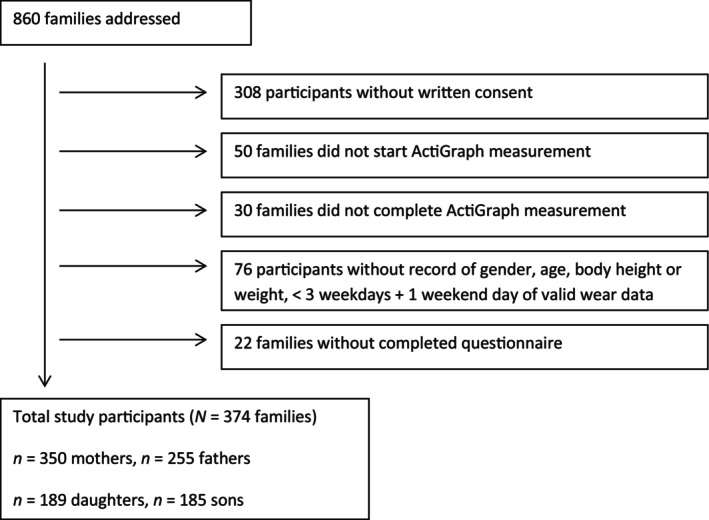
Illustration of participation selection.

Data were obtained from the Czech cross‐sectional FAMIly Physical Activity, Sedentary behaviour and Sleep (FAMIPASS) study (Sigmundova et al., 2023) that was established to evaluate lifestyle behaviours of families with at least one child aged between 3 and 8 years and to explore parent–child associations between segments of 24‐h movement behaviour (physical activity, sedentary behaviour, and sleep). A detailed description of FAMIPASS study has been published elsewhere (Sigmundova et al., 2023). A stratified random sample of participating families was recruited from selected kindergartens and primary schools from urban and rural parts of Bohemia, Moravia, and Silesia. From the 47 invited kindergartens and primary schools, 36 institutions provided consent to take part in the study (response rate = 76.6%). Detailed participation selection process including the exclusion procedure for the study can be viewed in Figure 1. The participating parents and children were predominantly Czech‐speaking White Caucasian (>96.5%), which corresponds to the very homogeneous ethnic demography of the Czech Republic (Bilik, 2018). Families (*n* = 374) in the final sample participated in the study on a voluntary basis. The target child was any child aged 3–8 years and all children in this age range were included in the analysis. Primary inclusion criteria consisted of an appropriate age of the child (children), sufficient health among all family members to enable participation, along with a willingness to monitor movement behaviours using accelerometer device for 7 days. Prior to the investigation, ethical approval was obtained from the Ethics Committee of the Faculty of Physical Culture, Palacký University Olomouc, Czech Republic (reference number: 25/2021).

### 2.2 Procedure

The data collection took place during the regular kindergarten/school days (not on holidays) between March 2022 and May 2023. After obtaining consent from educational institutions’ managements, a leaflet with a detailed description of the study was distributed to the potential participants. On receiving written informed consent for children's participation from parents interested in the study, the researchers, parents and kindergartens personnel attended an initial meeting where participants got familiar with the research process and measurements, and received individually set wrist‐worn accelerometers with instructions on how to wear the device and how to complete the daily log that included the information about the child's sleep, and the family logbook, a questionnaire that contained questions about the child's naptime and parent's sleep habits. Actigraphy for 7 days and the use of wrist‐worn devices, compared to those placed on hips, have been shown to provide reliable and acceptable measures of sleep (Lam et al., 2011; Scott et al., 2017).

The intent was to include the maximum number of family members (both parents and all children aged 3–8 years) feasible for each participating household; however, the family members involvement depended on the willingness to participate and the minimum number of accelerometers per family was two. The family size ranged from a minimum of two members (one parent and one child) to a maximum of six members (two parents and four children). To ensure accurate data collection and prevent device misplacement, each accelerometer was distinctly labelled with the participant's name and role (child or parent).

Researchers a week after, during a follow‐up meeting, collected the devices and family logbooks. Individual feedback and study results were distributed to the participating parents after completing data processing.

### 2.3 Accelerometry and sleep behaviour

All participants were instructed to wear an ActiGraph accelerometer (ActiGraph, Pensacola, FL, USA) on their non‐dominant wrist continuously for 24 h/day, over a period of 7 consecutive days and nights, excluding periods of bathing and swimming. Specifically, parents were equipped with the GT9x Link model, while children wore the wGT3x‐BT model.

Simultaneously, parents kept a 7‐day log that included the information about the child's waking times and bedtimes, physical activities, commuting to kindergarten/school, etc. (Sigmundova et al., 2023). The accelerometers were initialised to collect data with a 100 Hz‐sampling frequency using ActiLife software. To extract sleep behaviour parameters, the raw data were processed using the open‐source R package GGIR version 2.7–1, employing its default settings and the Heuristic algorithm looking at Distribution of Change in Z‐Angle (HDCZA) (Migueles et al., 2019; van Hees et al., 2018). Our study focused on three key sleep parameters: (i) Sleep time window (STW)—the duration between sleep onset and wake time; (ii) total sleep time (TST)—the aggregate time classified as sleep within the STW; and (iii) sleep percentage—this was calculated as the percentage of STW to TST, using the formula:

$$\left[\left(\mathrm{TST}/\mathrm{STW}\right)\times 100\right].$$

Accelerometer inclusion criteria consisted of obtaining a minimum of 3 weekdays and 1 weekend day of valid wear (≥16 h/day) and availability of wear data for each 15 min period in the 24‐h cycle (van Hees et al., 2018).

Sleep behaviours were assessed using information from the family logbooks, which parents completed daily. The questionnaire contained questions about bedtime routine of children including the use of screen media 2 h before bedtime, having a screen device in the bedroom, having own bedroom, and naptime and its duration, if applicable. Parent's sleep behaviours included questions about watching television (TV) before bedtime, the use of screen media before bedtime, working or studying in the bed, the use of alcohol/energy drinks (including coffee and black tea) before bedtime, and eating or smoking before bedtime or during the night. The response options to sleep questions were coded ‘yes or no’.

### 2.4 Sedentary behaviour and physical activity

To extract sedentary behaviour and physical activity parameters for participants, the raw data were processed using the open‐source R package GGIR version 2.7–1, applying age‐specific thresholds. This approach is detailed elsewhere (Migueles et al., 2019). Briefly, for both children and adults, sedentary behaviour and physical activity levels were determined based on milligravitational units (m*g*) from wake activities. Specifically, for children, activities <36 m*g* were classified as sedentary behaviour, between 36 and 200 m*g* as light physical activity, and >201 m*g* as moderate‐to‐vigorous physical activity (MVPA). In contrast, for adults, sedentary behaviour was classified as activities <45 m*g*, light physical activity ranged from 45 to 100 m*g*, and activities >101 m*g* were considered MVPA.

The criteria for meeting physical activity recommendations were defined as engaging in at least 180 min/day of total physical activity, with at least 60 min/day of MVPA for children aged 3–4 years, at least 60 min/day of MVPA for children aged ≥5 years, and at least 150 min of MVPA/week for adults (Paruthi et al., 2016). Sedentary behaviour in children was dichotomised as being less sedentary (≤444 min/day) versus being more sedentary (≥444 min/day) (Sigmundova et al., 2023).

### 2.5 Canadian sleep guidelines

Canadian 24‐h movement guidelines for children and adults were used for the determination of sleep habits and sleep guidelines adherence (CSEP, n.d.). According to these guidelines, preschoolers aged 3–4 years are recommended to get 10–13 h of good‐quality sleep (this may include a nap), children aged 5–13 years should have 9–11 h of uninterrupted sleep per night and adults are advised to get 7–9 h of good‐quality sleep on a regular basis (CSEP, n.d.). Nap times were included in the TST only in the children aged 3–4 years (*n* = 30) and parents were not given any specific information about the optimal sleep duration recommendation before the study. Participants were dichotomously classified as meeting the age‐specific sleep guidelines if their average STW fell within the recommended time intervals.

### 2.6 Statistical analysis

All statistical analyses were conducted using the IBM Statistical Package for the Social Sciences (SPSS), version 26 (IBM Corp). Descriptive data were generated for all analysed variables and baseline characteristics of participants can be found in Table 1. Logistic regression method backward stepwise (likelihood ratio) was used to examine mother–child/father–child relationship in sleep guideline recommendation adherence and the likelihood of meeting sleep guidelines for all analysed variables (Table 2). The regression parameters were based on the odds ratios (ORs) with 95% confidence intervals (CIs). Meeting sleep recommendation represented the dependent variables and analysed variables (i.e., screen time before sleep, screen media in bedroom, etc.) represented the independent variable. In the regression analysis one parent was represented multiple times in families with multiple children in the household that participated in the research (20.6% of participating families had more than one child included in the analyses). The independent‐samples Mann–Whitney *U* test measured differences in sleep percentage between boys and girls. An α level of *p* < 0.05 was set to determine significance.

**TABLE 1:** Characteristics of the study participants.

Characteristic	Total	Male	Female
Children	*N = 374*	*n* = 185	*n* = 189
Age, years, mean (SD)	6.5 (1.7)	6.6 (1.6)	6.4 (1.7)
Accelerometer valid days, mean (SD)	5.3 (0.6)	5.3 (0.6)	5.3 (0.7)
Non‐wear time, %, mean (SD)	1.6 (3.7)	1.5 (3.6)	1.79 (3.8)
Sleep guideline adherence, %^a^	65.5	60.0	70.9
STW, min/day, mean (SD)^b^	550.5 (52.6)	543.7 (53.9)	557.4 (50.32)
TST, min/day, mean (SD)^c^	449.0 (77.3)	445.7 (71.1)	452.3 (83.0)
Sleep percentage, %, mean (SD)^d^	81.1	81.4	77.1
Own bedroom, %	39.6	36.8	42.3
Screen media before bedtime, %^e^	74.1	73.5	74.6
Screen device in the bedroom, %	35.3	34.6	36.0
Parents	*N* = 615	*n* = 265	*n* = 350
Age, years, mean (SD)	37.4 (4.9)	39.7 (5.6)	36.8 (4.6)
Accelerometer valid days, mean (SD)	5.1 (0.5)	5.1 (0.6)	5.2 (0.6)
Non‐wear time, %, mean (SD)	1.5 (3.2)	1.7 (4.4)	1.3 (3.4)
Sleep guideline adherence, %^a^	58.3	52.4	64.3
STW, min/day, mean (SD)^b^	442.9 (96.5)	431.8 (101.6)	448.8 (94.2)
TST, min/day, mean (SD)^c^	376.8 (95.0)	360.6 (98.8)	389.2 (90.5)
Sleep percentage, %^d^	83.4	82.8	86.2
Higher education, %^f^	49.4	38.5	58.7
Mobile before bedtime, %^e^	83.4	84.9	84.7
TV before bedtime, %	73.3	80.7	70.0

*Note*: Data are presented as mean (standard deviation) for continuous variables and as percentages (%) for categorical variables.

^a^ Percentage of participants who met Canadian sleep recommendations.

^b^ Average time spent in sleep time window (STW).

^c^ Average time spent in total sleep time (TST).

^d^ The TST/STW × 100.

^e^ Percentage of children/parents who used screen media/mobile 2 h before bedtime, respectively.

^f^ Percentage of parents who graduated from university or community college.

**TABLE 2:** Correlates of meeting the Canadian sleep guideline recommendations in children.

Analysed variables	Mother–child model			Father–child model		
	*N*	OR	95% CI	*N*	OR	95% CI
PARENTS: Sleep recommendation adherence						
No	125	Ref.			NS	
Yes	225	1.72*	1.06–2.79			
Higher education						
No	147	Ref.			NS	
Yes	203	0.60*	0.36–0.99			
CHILDREN:						
Gender						
Boys	172	Ref.			NS	
Girls	178	1.85*	1.15–2.97			
Sedentary behaviour						
Less sedentary^a^	173	Ref.		134	Ref.	
More sedentary^b^	177	0.52**	0.32–0.83	121	0.48**	0.29–0.82
Screen device in the bedroom						
Yes	123	Ref.		93	Ref.	
No	227	2.11**	1.26–3.53	162	2.07**	1.21–3.54

Abbreviations: CI, confidence interval; *N*, number of participants; NS, non‐significant results for variables; OR, odd ratio for each variable, logistic regression method backward stepwise (likelihood ratio); Ref., reference group.

^a^ <444 min/day.

^b^ ≥444 min/day.

* *p* < 0.05;

** *p* < 0.01.

## 3 RESULTS

The descriptive characteristics of participants including the list of analysed variables are shown in Table 1. In children, duration in STW (*p* = 0.009) and duration of TST (*p* = 0.018) were significantly higher in girls, compared to boys. The girls and boys slept for a mean (standard deviation [SD]) of 557.4 (50.32) and 543.7 (53.9) min/day, respectively. The mean (SD) time spent in TST in girls and boys was 452.3 (83.0) and 445.7 (71.1) min/day, respectively. Moreover, the data revealed that children who met sleep recommendations slept significantly (*p* < 0.001) longer (STW: mean [SD] 578.5 [28.5] min/day) and spent significantly (*p* < 0.001) more time in TST (mean [SD] 476.1 [59.1] min/day), compared to children who did not meet the sleep recommendations (STW: mean [SD] 497.6 [46.6] min/day, TST: mean [SD] 397.8 [81.7] min/day). Also, children who fulfilled the sleep recommendations were ~4 months younger (*p* < 0.04) (mean [SD] age 6.4 [1.6] years) than those who did not (mean [SD] age 6.7 [1.7] years) (data not shown).

Table 2 presents an overview of correlates of meeting the Canadian sleep recommendations in children. Children were more likely to meet the sleep guidelines if their mother (but not father) fulfilled the recommended amount of sleep (*p* < 0.05) and did not have a degree from university or community college (*p* < 0.05). Furthermore, significantly higher odds of meeting the required sleep amount in children were related with female gender (*p* < 0.05), not having a screen device in the bedroom (*p* < 0.01) and being less sedentary (*p* < 0.05) (Table 2). No significant associations were found between children's sleep guidelines adherence and following variables in the mother–child and father–child models—children: weight status, health status, fitness, having own bedroom, using screen 2 h before bedtime; parents: using mobile/TV before bedtime.

The only significant variables affecting the odds of meeting sleep guidelines in parents were being overweight/obese (father's model, OR 0.46, 95% CI 0.27–0.78; *p* < 0.01) and completing 150 min/week of recommended physical activity (mother's model, OR 1.78, 95% CI 1.01–3.17; *p* < 0.05). No significant relationships were observed in meeting the sleep guidelines of parents and other analysed variables (both parents: education, screen media or mobile phones before bedtime, sedentary job, meal or alcohol before sleeping; mothers: weight status; fathers: fulfilling recommended physical activity).

## 4 DISCUSSION

The present study investigated the accelerometer‐assessed sleep behaviours and associations in parent–child sleep guideline compliance and sleep quality in a sample of 374 Czech families with children aged 3–8 years. To the best of our knowledge, this is the first study examining sleep behaviours within a family context in preschoolers.

The findings of this study revealed that 65.5% of children (60% boys, 70.9% girls) and 58.3% of parents (52.4% fathers, 64.3% mothers) achieved the recommended sleep duration. The most recent studies that addressed sleep guideline adherence in preschool aged children were a part of a research focusing on meeting the 24‐h movement guidelines in three behaviours (sleep, physical activity, and sedentary behaviour; Decraene et al., 2021; Fairclough et al., 2023; Kim et al., 2020; Kracht et al., 2019; Tapia‐Serrano et al., 2022). According to these studies, the sleep length, compared to physical activity and sedentary behaviour, had the greatest adherence rate in young children (Decraene et al., 2021). A cross‐sectional study examining the proportion of preschoolers who met the 24‐h movement behaviour in six European countries (Belgium, Bulgaria, Germany, Greece, Poland, and Spain) found that 69.2% of preschoolers achieved the recommended sleep duration (Decraene et al., 2021), which was in concordance with our findings. Higher sleep duration prevalence rates were reported in some studies with participants from Japan (82.5%; Kim et al., 2020) or the USA (86.9%; Kracht et al., 2019). The comparison of sleep prevalence rates from different studies should be taken with caution because of the differences in sleep assessment tools used, which may cause the variability in obtained data. In a recent review, Tapia‐Serrano et al. (2022) found that 20 studies assessed sleep duration by device‐based measures and other studies used questionnaires (26 self‐reported and 17 parent‐reported). Even though sleep prevalence rates in our findings are consistent with other European countries (Decraene et al., 2021), it is far from ideal, especially when considering age‐related differences in sleep (e.g., 71% of children aged 8–13 years and 75.3% of adolescents from the Czech Republic were not able to sleep >9 and 8 h/day, respectively; Fairclough et al., 2023; Gaba et al., 2020) and the decrease in trends in sleep duration over time (Matricciani et al., 2012).

Our results also found that mothers and daughters had significantly greater quantity of sleep, compared to fathers and sons. These results are not consistent with most of the studies (Decraene et al., 2021; Kim et al., 2020; Kracht et al., 2019); however, few studies found significant gender‐related differences in sleep length in children (Fairclough et al., 2023). A possible explanation for our finding might be that the greater time spent in sleep in females is due to the compensatory change between behaviours, especially relocating time to sleep from physically active and sedentary time. Studies on variations in accelerometer‐assessed behaviours in European countries documented girls aged 2–10 years were less active and more sedentary, compared to boys (Steene‐Johannessen et al., 2020), which may support this explanation.

Children who met sleep recommendations also spent significantly more time in the TST phase, compared to children who did not follow the sleep recommendations and more females than males spent sufficient time in TST. Uninterrupted sleep is linked with greater sleep quality and diminishes numerous health consequences (e.g., disruption of the sleep cycle, daytime sleepiness, mood disturbances, etc.; Van Someren et al., 2015; Wilckens et al., 2014), and therefore, might be associated with longer sleep duration and compliance with sleep guidelines. As longer sleep was documented more frequently in female gender, it is not surprising that time in TST was higher in mothers and daughters, compared to males, which has been supported by literature (Sadeh et al., 2000). Age‐appropriate sleep quantity and quality is affected by the support and promotion of healthy sleep behaviours in the family (Rhodes et al., 2020) that include parents’ involvement in establishing bedtime rules and endorsing sleep hygiene (Buxton et al., 2015).

Related to parent–child relationship, in our sample, preschoolers were more likely to meet the sleep guidelines if their mother (but not father) fulfilled the required amount of sleep. These results agree with the findings of previous studies evaluating parent–child associations, in which it has been reported that children sleep is connected to the sleep behaviours of their parents and this relationship also continues to be relevant during adolescent years (Fuligni et al., 2015; Varma et al., 2021). Based on the current evidence, the accordance in sleep–wake patterns between parents and children has been shown to be ~70% (Varma et al., 2022) and this interrelation tends to be bidirectional between mothers and children, e.g., children's sleep affects the sleep of their mothers and vice versa (Varma et al., 2021). The prediction between the mother's and child's sleep (but not the father's and child's) behaviour has been well established in both cross‐sectional and longitudinal studies (Kouros & El‐Sheikh, 2017; Varma et al., 2021). In the family, mothers and fathers play distinct roles in parenting (Pakaluk & Price, 2020; Yaffe, 2020). Mothers compared to fathers spend more time interacting with their children, are more involved in childcare, and are more responsive to the child's needs, which could explain the significant relationship only in mother–child dyads (Pakaluk & Price, 2020; Yaffe, 2020). In addition, only a limited number of studies examined sleep relations between fathers and children and the few studies that addressed both parents–child sleep associations included more females than males (Varma et al., 2021; Varma et al., 2022), which could be the reason why the significant sleep relationship is found only in mothers and children. Future studies should focus on recruiting more fathers to further establish associations between father–child dyads.

Significantly higher odds of children meeting the required sleep duration were related with mothers’ (lower education level) and children's (female gender, being less sedentary and not having a screen device in the bedroom) factors. In contrast with earlier findings that documented the positive association between parental education level and child sleep behaviour, especially in mothers (Bøe et al., 2012; McDowall et al., 2017), higher education level of mothers in our sample was a predictor of noncompliance with sleep guidelines in children. The result may be explained by the fact that some mothers with lower levels of education might be working at night and as mothers are considered to be primary caregivers to their children, they might facilitate earlier bedtimes before going to work. It is important to note, that the present research data did not include information concerning parent employment and shift work, and this explanation is only speculative. Another important factor that has been associated with children's sleep recommendation adherence include screen behaviour (Hale et al., 2018). Our results support previous research indicating that having a screen‐media device in the bedroom is linked with shorter sleep (Hale et al., 2018); however, this study has been unable to confirm the relationship between sufficient sleep duration and other screen behaviours (using screen devices before bedtime, etc.). As screen time in children has been significantly associated with parent–child interaction (Foo et al., 2022), it is important to educate parents and their children on sleep‐friendly screen practices.

### 4.1 Strengths and limitations

The strengths of the present study include a large representative sample of the families with preschoolers, objectively measured sleep screening, high response rates, data from all three Czech regions, verified study protocol, and examining both the mother–child and father–child relationship in sleep guideline adherence. Study limitations that should be taken into consideration are as follows: (i) a cross‐sectional design did not allow evidence for establishing a causal relationship between parent–child sleep guideline adherence; (ii) the study neither investigated associations between correlates separately for weekdays and weekends nor included all sleep variables that could be related to sleep recommendation compliance; (iii) ~20% of the families had multiple children involved in the study, resulting in one parent being included multiple times in the analyses examining the parent–child relationship in adherence to sleep recommendations. However, a subgroup sensitivity analysis indicated no significant differences in the ORs between groups when a parent was included multiple times versus just once.

## 5 CONCLUSION

The present study revealed that 34.5% of children aged 3–8 years and 41.7% of parents did not meet the Canadian guideline recommendation for sleep, and significantly more girls/mothers, compared to boys/fathers, complied with these guidelines, and spent sufficient time in TST. Moreover, the sleep duration in mothers and children was found to be interrelated and meeting the sleep recommendations in children was related to female gender, the absence of screen device in the bedroom, spending ≤444 min/day sedentary and having a mother with a lower education level. These findings provided information about current moderate‐to‐high prevalence rates in sleep guideline noncompliance in preschoolers and their parents, which could contribute to numerous health consequences. Because of the found relationship between the sleep behaviours of mothers and their children, it is important to promote healthy sleep behaviours in the whole family. Monitoring of the sleep behaviours in preschoolers should be continued to provide the evidence and support for future interventions. Future studies should focus on the comparison of sleep guideline adherence separately for weekdays and weekends, examination of the associations between father–child sleep behaviours or monitoring of other variables related to sleep such as sleep jet lag, sleep efficiency or wake times/bedtimes.

## AUTHOR CONTRIBUTIONS

**Jaroslava Voráčová:** Writing – original draft; writing – review and editing; validation. **Erik Sigmund:** Conceptualization; funding acquisition; writing – review and editing. **Michal Vorlíček:** Methodology; validation; writing – review and editing. **Jan Dygrýn:** Methodology; data curation; writing – review and editing; validation. **Dagmar Sigmundová:** Conceptualization; formal analysis; funding acquisition; writing – review and editing.

## FUNDING INFORMATION

This study was supported by the Czech Science Foundation, grant number 22‐22765S. The described study is from the project ‘Research of Excellence on Digital Technologies and Wellbeing CZ.02.01.01/00/22_008/0004583’, which is co‐financed by the European Union.

## CONFLICT OF INTEREST STATEMENT

The authors declare that they have no conflict of interest.

## Data Availability

The data that support the findings of this study will be available upon reasonable request after the completion of the project.

## References

[jsr14242-bib-0001] Batool, S. H. , Safdar, M. , & Eman, S. (2024). Relationship between parents' health literacy and child health: Systematic review. Library Hi Tech, 42(1), 131–148.

[jsr14242-bib-0002] Bilik, J . (Ed.). (2018). Demographic yearbook of districts of The Czech Republic – 2008–2017 (1st ed., pp. 1–145). Czech Statistical Office.

[jsr14242-bib-0003] Bøe, T. , Hysing, M. , Stormark, K. M. , Lundervold, A. J. , & Sivertsen, B. (2012). Sleep problems as a mediator of the association between parental education levels, perceived family economy and poor mental health in children. Journal of Psychosomatic Research, 73(6), 430–436. 23148810 10.1016/j.jpsychores.2012.09.008

[jsr14242-bib-0004] Buxton, O. M. , Chang, A. M. , Spilsbury, J. C. , Bos, T. , Emsellem, H. , & Knutson, K. L. (2015). Sleep in the modern family: Protective family routines for child and adolescent sleep. Sleep Health, 1(1), 15–27. 26779564 10.1016/j.sleh.2014.12.002 PMC4712736

[jsr14242-bib-0005] Chaput, J. P. , Dutil, C. , Featherstone, R. , Ross, R. , Giangregorio, L. , Saunders, T. J. , Janssen, I. , Poitras, V. J. , Kho, M. E. , Ross‐White, A. , & Carrier, J. (2020). Sleep duration and health in adults: An overview of systematic reviews. Applied Physiology, Nutrition, and Metabolism, 45(10 (Suppl. 2)), S218–S231. 10.1139/apnm-2020-0034 33054337

[jsr14242-bib-0006] Chaput, J. P. , Gray, C. E. , Poitras, V. J. , Carson, V. , Gruber, R. , Olds, T. , Weiss, S. K. , Connor Gorber, S. , Kho, M. E. , Sampson, M. , Belanger, K. , Eryuzlu, S. , Callender, L. , & Tremblay, M. S. (2016). Systematic review of the relationships between sleep duration and health indicators in school‐aged children and youth. Applied Physiology, Nutrition, and Metabolism, 41(6 (Suppl. 3)), S266–S282. 10.1139/apnm-2015-0627 27306433

[jsr14242-bib-0007] Cimon‐Paquet, C. , Tetreault, E. , & Bernier, A. (2019). Early parent‐child relationships and child sleep at school age. Journal of Applied Developmental Psychology, 64, 101057.

[jsr14242-bib-0008] Corepal, R. , Tully, M. A. , Kee, F. , Miller, S. J. , & Hunter, R. F. (2018). Behavioural incentive interventions for behaviour change in young people (5–18 years old): A systematic review and meta‐analysis. Preventive Medicine, 110, 55–66. 29432789 10.1016/j.ypmed.2018.02.004

[jsr14242-bib-0009] CSEP Canadian 24‐hour movement guidelines: An integration of physical activity, sedentary behaviour, and sleep. Ontario: The Canadian Society for Exercise Physiology, 2021. Available at: https://csepguidelines.ca [cit. 2023‐11‐13]

[jsr14242-bib-0010] Decraene, M. , Verbestel, V. , Cardon, G. , Iotova, V. , Koletzko, B. , Moreno, L. A. , Miguel‐Berges, M. L. , Gurzkowska, B. , Androutsos, O. , Manios, Y. , & de Craemer, M. (2021). Compliance with the 24‐hour movement behaviour guidelines and associations with adiposity in European pre‐schoolers: Result from the ToyBox‐study. Environmental Research and Public Health, 18, 7499. 34299954 10.3390/ijerph18147499 PMC8307342

[jsr14242-bib-0011] El‐Sheikh, M. , & Sadeh, A. I. (2015). Sleep and development: Introduction to the monograph. Monographs of the Society for Research in Child Development, 80(1), 1–14. 10.1111/mono.12141 25704732

[jsr14242-bib-0012] Fairclough, S. J. , Clifford, L. , Brown, L. , & Tyler, R. (2023). Characteristics of 24‐hour movement behaviours and their associations with mental health in children and adolescents. Journal of Activity, Sedentary and Sleep Behaviors, 2(1), 1–14. 10.1186/s44167-023-00021-9 PMC10234795 38013786

[jsr14242-bib-0013] Foo, N. , Ismail, N. , & Arshat, Z. (2022). Relationship between screen time, sleep duration, parent‐child interaction and psychosocial adjustment among preschool children in Selangor, Malaysia. Journal of Educational and Developmental Psychology, 12, 94.

[jsr14242-bib-0014] Fuligni, A. J. , Tsai, K. M. , Krull, J. L. , & Gonzales, N. A. (2015). Daily concordance between parent and adolescent sleep habits. Journal of Adolescent Health, 56(2), 244–250. 10.1016/j.jadohealth.2014.09.013 PMC4306808 25620309

[jsr14242-bib-0015] Gaba, A. , Dygryn, J. , Stefelova, N. , Rubín, L. , Hron, K. , Jakubec, L. , & Pedišić, Ž. (2020). How do short sleepers use extra waking hours? A compositional analysis of 24‐h time‐use patterns among children and adolescents. International Journal of Behavioural Nutrition and Physical Activity, 17, 104. 10.1186/s12966-020-01004-8 PMC7427741 32795287

[jsr14242-bib-0016] Gariepy, G. , Danna, S. , Gobing, I. , Rasmussen, M. , de Matos, M. G. , Tynjälä, J. , Janssen, I. , Kalman, M. , Villeruša, A. , Husarova, D. , Brooks, F. , Elgar, F. J. , Klavina‐Makrecka, S. , Šmigelskas, K. , Gaspar, T. , & Schnohr, C. (2020). How are adolescents sleeping? Adolescent sleep patterns and sociodemographic differences in 24 European and north American countries. Journal of Adolescent Health, 66(6), S81–S88. 10.1016/j.jadohealth.2020.03.013 32446613

[jsr14242-bib-0017] Hale, L. , Kirschen, G. W. , LeBourgeois, M. K. , Gradisar, M. , Garrison, M. M. , Montgomery‐Downs, H. , Kirschen, H. , McHale, S. M. , Chang, A.‐M. , & Buxtonj, O. M. (2018). Youth screen media habits and sleep: Sleep‐friendly screen‐behavior recommendations for clinicians, educators, and parents. Child and Adolescent Psychiatric Clinics of North America, 27(2), 229–245. 29502749 10.1016/j.chc.2017.11.014 PMC5839336

[jsr14242-bib-0018] Kim, H. , Ma, J. , Harada, K. , Lee, S. , & Gu, Y. (2020). Associations between adherence to combinations of 24‐h movement guidelines and overweight and obesity in Japanese preschool children. Environmental Research and Public Health, 17, 9320. 33322103 10.3390/ijerph17249320 PMC7763194

[jsr14242-bib-0019] Kouros, C. D. , & El‐Sheikh, M. (2017). Within‐family relations in objective sleep duration, quality, and schedule. Child Development, 88(6), 1983–2000. 27859005 10.1111/cdev.12667 PMC5425327

[jsr14242-bib-0020] Kracht, C. L. , Webster, E. K. , & Staiano, A. E. (2019). Sociodemographic differences in young children meeting 24‐hour movement guidelines. Journal of Physical Activity and Health, 16, 908–915. 31491748 10.1123/jpah.2019-0018 PMC7058481

[jsr14242-bib-0021] Lam, J. C. , Mahone, E. M. , Thornton, B. , & Scharf, S. M. (2011). Defining the roles of actigraphy and parent logs for assessing sleep variables in preschool children. Behavioral Sleep Medicine, 9(3), 184–193. 21722013 10.1080/15402002.2011.583906 PMC3206727

[jsr14242-bib-0022] Matricciani, L. , Bin, Y. S. , Lallukka, T. , Kronholm, E. , Dumuid, D. , Paquet, C. , & Olds, T. (2017). Past, present, and future: Trends in sleep duration and implications for public health. Sleep Health, 3(5), 317–323. 28923186 10.1016/j.sleh.2017.07.006

[jsr14242-bib-0023] Matricciani, L. , Olds, T. , & Petkov, J. (2012). In search of lost sleep: Secular trends in the sleep time of school‐aged children and adolescents. Sleep Medicine Review, 16(3), 203–211. 10.1016/j.smrv.2011.03.005 21612957

[jsr14242-bib-0024] McDowall, P. S. , Elder, D. E. , & Campbell, A. J. (2017). Relationship between parent knowledge of child sleep, and child sleep practices and problems: A pilot study in a children's hospital cohort. Journal of Paediatrics and Child Health, 53(8), 788–793. 28425627 10.1111/jpc.13542

[jsr14242-bib-0025] Migueles, J. H. , Rowlands, A. V. , Huber, F. , Sabia, S. , & van Hees, V. T. (2019). GGIR: A research community–driven open source R package for generating physical activity and sleep outcomes from multi‐day raw accelerometer data. Journal for the Measurement of Physical Behaviour, 2(3), 188–196.

[jsr14242-bib-0026] Miller, M. A. , Bates, S. , Ji, C. , & Cappuccio, F. P. (2020). Systematic review and meta‐analyses of the relationship between short sleep and incidence of obesity and effectiveness of sleep interventions on weight gain in preschool children. Obesity Reviews, 22(2), e13113. 33237635 10.1111/obr.13113

[jsr14242-bib-0027] Niec, L. N. , Todd, M. , Brodd, I. , & Domoff, S. E. (2022). PCIT‐health: Preventing childhood obesity by strengthening the parent‐child relationship. Cognitive and Behavioural Practice, 29(2), 335–347.

[jsr14242-bib-0028] Pakaluk, C. R. , & Price, J. (2020). Are mothers and fathers interchangeable caregivers? Marriage & Family Review, 56(8), 784–793.

[jsr14242-bib-0029] Paruthi, S. , Brooks, L. J. , D'Ambrosio, D. , Hall, W. A. , Kotagal, S. , Lloyd, R. M. , Malow, B. A. , Maski, K. , Nichols, C. , Quan, S. F. , Rosen, C. L. , Troester, M. M. , & Wise, M. S. (2016). Consensus statement of the American Academy of sleep medicine on the recommended amount of sleep for healthy children: Methodology and discussion. Journal of Clinical Sleep Medicine, 12(11), 1549–1561. 27707447 10.5664/jcsm.6288 PMC5078711

[jsr14242-bib-0030] Philbrook, L. E. , Aguilar, K. , Bohan, A. R. , Daza, K. M. , & Harris, S. L. (2022). Bedtime parenting practices and sensitivity are associated with young children's sleep. Journal of Family Psychology, 36(8), 1473–1479. 36037504 10.1037/fam0001027

[jsr14242-bib-0031] Quist, J. S. , Sjodin, A. , Chaput, J. P. , & Hjorth, M. F. (2016). Sleep and cardiometabolic risk in children and adolescents. Sleep Medicine Reviews, 29, 76–100. 26683701 10.1016/j.smrv.2015.09.001

[jsr14242-bib-0032] Rhodes, R. E. , Guerrero, D. , & Vanderloo, L. M. (2020). Development of a consensus statement on the role of the family in the physical activity, sedentary, and sleep behaviours of children and youth. International Journal of Behavioural Nutrition and Physical Activity, 17, 74. 10.1186/s12966-020-00973-0 PMC7296673 32539730

[jsr14242-bib-0033] Rollo, S. , Antsygina, O. , & Tremblay, M. S. (2020). The whole day matters: Understanding 24‐hour movement guideline adherence and relationships with health indicators across the lifespan. Journal of Sport and Health Science, 9(6), 493–510. 32711156 10.1016/j.jshs.2020.07.004 PMC7749249

[jsr14242-bib-0034] Sadeh, A. , Raviv, A. , & Gruber, R. (2000). Sleep patterns and sleep disruptions in school‐age children. Developmental Psychology, 36(3), 291–301. 10830974 10.1037//0012-1649.36.3.291

[jsr14242-bib-0035] Scott, J. J. , Rowlands, A. V. , Cliff, D. P. , Morgan, P. J. , Plotnikoff, R. C. , & Lubans, D. R. (2017). Comparability and feasibility of wrist‐ and hip‐worn accelerometers in free‐living adolescents. Journal of Science and Medicine in Sport, 20(12), 1101–1106. 28501418 10.1016/j.jsams.2017.04.017

[jsr14242-bib-0036] Sheenan, C. M. , Frochen, S. E. , Walsemann, K. M. , & Ailshire, J. A. (2019). Are U.S. adults reporting less sleep?: Findings from sleep duration trends in the National Health Interview Survey, 2004–2017. Sleep, 42(2), zsy221. 30452725 10.1093/sleep/zsy221 PMC6941709

[jsr14242-bib-0037] Short, M. A. , Blunden, S. , Rigney, G. , Matricciani, L. , Coussens, S. , M. Reynolds, C. , & Galland, B. (2018). Cognition and objectively measured sleep duration in children: A systematic review and meta‐analysis. Sleep Health, 4(3), 292–300. 29776624 10.1016/j.sleh.2018.02.004

[jsr14242-bib-0038] Sigmund, E. , Sigmundova, D. , & Badura, P. (2020). Excessive body weight of children and adolescents in the spotlight of their parents' overweight and obesity, physical activity, and screen time. International Journal of Public Health, 65, 1309–1317. 32613262 10.1007/s00038-020-01419-x PMC7588386

[jsr14242-bib-0039] Sigmundova, D. , Dygryn, J. , Vorlicek, M. , Banátová, K. , Voráčová, J. , & Sigmund, E. (2023). FAMILy physical activity, sedentary behaviour and sleep (FAMIPASS) study: Protocol for a cross‐sectional study. BMJ Open, 13, e073244. 10.1136/bmjopen-2023-073244 PMC10407347 37550023

[jsr14242-bib-0040] Sigmundova, D. , & Sigmund, E. (2021). Weekday‐weekend sedentary behaviour and recreational screen time patterns in families with preschoolers, schoolchildren, and adolescents: Cross‐sectional three cohort study. International Journal of Environmental Research and Public Health, 18(9), 4532. 33923313 10.3390/ijerph18094532 PMC8123206

[jsr14242-bib-0041] Steene‐Johannessen, J. , Hansen, B. H. , Dalene, K. E. , Kolle, E. , Northstone, K. , Møller, N. C. , Grøntved, A. , Wedderkopp, N. , Kriemler, S. , Page, A. S. , Puder, J. J. , Reilly, J. J. , Sardinha, L. B. , van Sluijs, E. M. F. , Andersen, L. B. , van der Ploeg, H. , Ahrens, W. , Flexeder, C. , Standl, M. , … Cardon, G. (2020). Variations in accelerometry measured physical activity and sedentary time across Europe – Harmonized analyses of 47,497 children and adolescents. International Journal of Behavioural Nutrition and Physical Activity, 17, 38. 10.1186/s12966-020-00930-x PMC7079516 32183834

[jsr14242-bib-0042] Tapia‐Serrano, M. A. , Sevil‐Serrano, J. , Sánchez‐Miguel, P. A. , López‐Gil, J. F. , Tremblay, M. S. , & García‐Hermoso, A. (2022). Prevalence of meeting 24‐hour movement guidelines from pre‐school to adolescence: A systematic review and meta‐analysis including 387,437 participants and 23 countries. Journal of Sport and Health Science, 11(4), 427–437. 35066216 10.1016/j.jshs.2022.01.005 PMC9338333

[jsr14242-bib-0043] Tikotzky, L. (2017). Parenting and sleep in early childhood. Current Opinion in Psychology, 15, 118–124. 28813250 10.1016/j.copsyc.2017.02.016

[jsr14242-bib-0044] van Hees, V. T. , Sabia, S. , Jones, S. E. , Wood, A. R. , Anderson, K. N. , Kivimäki, M. , Frayling, T. M. , Pack, A. I. , Bucan, M. , Trenell, M. I. , Mazzotti, D. R. , Gehrman, P. R. , Singh‐Manoux, B. A. , & Weedon, M. N. (2018). Estimating sleep parameters using an accelerometer without sleep diary. Scientific Reports, 8(1), 12975. 30154500 10.1038/s41598-018-31266-z PMC6113241

[jsr14242-bib-0045] Van Someren, E. J. , Cirelli, C. , Dijk, D. J. , Van Cauter, E. , Schwartz, S. , & Chee, M. W. L. (2015). Disrupted sleep: From molecules to cognition. The Journal of Neuroscience: The Official Journal of the Society for Neuroscience, 35(41), 13889–13895. 26468189 10.1523/JNEUROSCI.2592-15.2015 PMC4604227

[jsr14242-bib-0046] Varma, P. , Conduit, R. , Junge, M. , Lee, V. V. , & Jackson, M. L. (2021). A systematic review of sleep associations in parents and children. Journal of Child and Family Studies, 30, 2276–2288.

[jsr14242-bib-0047] Varma, P. , Jackson, M. , & Junge, M. (2022). Actigraphy‐measured sleep concordance, night‐wakings, intraindividual sleep variability in parents and their children – Associations with childhood sleep disturbances. Journal of Sleep Research, 32(3), e13773. 36345126 10.1111/jsr.13773

[jsr14242-bib-0048] WHO Guidelines on physical activity, sedentary behaviour and sleep for children under 5 years of age. World Health Organization, 2019. Available at: https://iris.who.int/handle/10665/311664. [cit. 2024‐1‐12] 31091057

[jsr14242-bib-0049] Wilckens, K. A. , Woo, S. G. , Kirk, A. R. , Erickson, K. I. , & Wheeler, M. E. (2014). Role of sleep continuity and total sleep time in executive function across the adult lifespan. Psychology and Aging, 29(3), 658–665. 25244484 10.1037/a0037234 PMC4369772

[jsr14242-bib-0050] Yaffe, Y. (2020). Systematic review of the differences between mothers and fathers in parenting styles and practices. Current Psychology, 42, 16011–16024.

